# Genotypic analysis of Shiga toxin-producing Escherichia coli clonal complex 17 in England and Wales, 2014–2022

**DOI:** 10.1099/jmm.0.001928

**Published:** 2024-11-07

**Authors:** Ching-Ying J. Poh, Ella V. Rodwell, Gauri Godbole, Claire Jenkins

**Affiliations:** 1 Gastrointestinal Bacteria Reference Unit, UK Health Security Agency, Colindale, London, UK; 2 NIHR Health Protection Research Unit in Gastrointestinal Infections, University of Liverpool, Liverpool, UK; 3 Gastro and Food Safety (One Health) Division, UK Health Security Agency, Colindale, London, UK

**Keywords:** epidemiology, genome sequence, molecular microbiology, Shiga toxin-producing *Escherichia coli* O103:H2, surveillance

## Abstract

**Introduction.** Shiga toxin-producing *Escherichia coli* (STEC) are zoonotic, gastrointestinal pathogens characterized by the presence of the Shiga toxin (*stx*) gene. Historically, STEC O157:H7 clonal complex (CC) 11 has been the most clinically significant serotype; however, recently there has been an increase in non-O157 STEC serotypes, including STEC O103:H2 belonging to CC17.

**Gap statement.** STEC O103:H2 is an STEC serotype frequently isolated in England, although little is known about the epidemiology, clinical significance, associated public health burden or evolutionary context of this strain.

**Aim.** Surveillance data and whole-genome sequencing data were analysed to determine the microbiological characteristics and public health burden of CC17, including the clinically significant serotype O103:H2, in England and Wales.

**Methodology.** Isolates of *E. coli* belonging to CC17 (*n*=425) submitted to the Gastrointestinal Bacteria Reference Unit from 2014 to 2022 were whole genome sequenced, integrated with enhanced surveillance questionnaire data and analysed retrospectively.

**Results.** Overall, diagnoses of CC17 infection increased every year since 2014. Most cases were female (58.5%), with the highest proportion of cases belonging to the 0–4 age group (*n*=83/424, 19.6%). Clinical presentation data identified diarrhoea (92.1%), abdominal pain (72.4%) and blood in stool (55.3%) as the most frequent symptoms, while 20.4% cases were admitted to hospital and 1.3% developed haemolytic uraemic syndrome. The five most common established serotypes were O103:H2 (64.5%), O123:H2 (11.1%), O151:H2 (6.6%), O71:H2 (3.3%) and O4:H2 (2.6%). The majority of CC17 isolates (78.6%) had the *stx1a/eae* virulence gene combination. Nine outbreak clusters of STEC infections that were mainly geographically dispersed and temporally related were identified and associated with foodborne transmission.

**Conclusions.** Nationwide implementation of PCR to detect non-O157 STEC and improvements to algorithms for the follow-up of PCR-positive faecal specimens is recommended. Enhanced surveillance is necessary to assess the incidence of CC17 infection and overall burden of this CC within the UK population.

## Introduction

Pathogenic *Escherichia coli* that cause symptoms of gastrointestinal (GI) disease are referred to as diarrheagenic *E. coli* (DEC) [1]. Shiga toxin-producing *E. coli* (STEC) is a zoonotic DEC pathotype that causes outbreaks of foodborne GI infection every year, most often in late summer and early autumn [2,3]. Most GI infections are mild and self-limiting and require minimal clinical intervention; however, STEC infections are associated with more severe clinical outcomes and are considered a public health priority. Common clinical symptoms of STEC include abdominal pain and bloody diarrhoea, and infection can lead to the development of haemolytic uraemic syndrome (HUS), which is the leading cause of acute kidney failure in young children in the UK and can be fatal [4].

The main animal reservoir of STEC is ruminants, and transmission from animals to humans occurs via direct contact with animals or their environment, or via the consumption of contaminated food or water [3,5]. STEC has a low infectious dose, which also facilitates person-to-person spread, and household transmission and outbreaks in nursery schools are common. STEC are characterized by the ability to produce Shiga toxin (Stx) [1]. Following infection, toxin release results in inhibition of protein synthesis and impaired cell function, damaging the intestinal epithelium and releasing *stx* into the bloodstream, causing damage to the kidneys [4]. There are two types of *stx* encoded by *stx1* and *stx2*, and at least ten well established *stx* subtypes (*stx1a, stx1c* and *stx1d* and *stx2a–stx2g*). *Stx2a*, *stx2c* and *stx2d* are associated with more severe disease manifestations [6]. Other virulence factors include the protein intimin, encoded by the *eae* gene, which is located on the locus of enterocyte effacement (LEE) [6].

There are over 400 serotypes of STEC, and historically in the UK, the most common was serotype O157:H7, clonal complex (CC) 11 [5]. However, over the past decade, there has been a decrease in the incidence of O157 STEC cases in England and an increase in non-O157 STEC cases [2,3]. Previous studies in the UK investigated the occurrence of non-O157 STEC of these serotypes as one group [2,7]. We now recognize there is a need to consider each serotype independently to determine the pathogenic potential to inform clinical management and assess the risk to public health. Globally, STEC O103:H2, belonging to CC17, is one of the most frequently reported serotypes and was classed as one of the ‘big six’ serogroups by the US Food and Drug Administration [8,9]. In the UK, STEC O103 was identified as the fifth most prevalent non-O157 serogroup and had the second highest frequency of bloody diarrhoea after STEC O157 [2].

Despite the increasing clinical importance of the STEC O103:H2, the literature provides little information regarding the epidemiological context and genomic complexity of this serotype in the UK. The aim of this study was to integrate genomic data with epidemiological data to gain further insight into the clinical and public health burden of O103:H2 and CC17 in England and Wales.

## Methods

### Microbiology

In England and Wales, faecal specimens collected from hospitalized patients and patients in the community presenting to their General Practitioner with symptoms of gastroenteritis are tested against a panel of GI pathogens (e.g. *Shigella* spp., STEC, *Salmonella* spp., *Vibrio* spp., and *Campylobacter* spp.) at local hospital diagnostic laboratories [10]. In diagnostic laboratories, cefixime tellurite sorbitol MacConkey (CT-SMAC) agar and/or commercial *E. coli* O157 antisera is used for the culture and isolation of non-sorbitol fermenting colonies that are characteristic of STEC O157, which are then referred to the Gastrointestinal Bacteria Reference Unit (GBRU) at the UK Health Security Agency (UKHSA) for confirmation and typing. Where local laboratories have implemented commercial molecular GI PCR panels, faecal specimens detected to be positive for the *stx* gene can be referred directly to the GBRU for confirmatory PCR testing and culture. Alternatively, where able, local laboratories may culture the *stx*-positive faecal specimens onto CT-SMAC and STEC chromogenic agar for the isolation of presumptive STEC for referral to GBRU as an isolate [11].

Within GBRU, all faecal specimens requesting STEC testing are inoculated into tryptone soya broths (Oxoid) and cultured onto MacConkey agar, sorbitol MacConkey agar and Colorex STEC chromogenic agar (E and O Laboratories) for the detection and isolation of individual colonies tested using in-house PCR assays targeting *stx1*, *stx2*, *eae* and *rfbEO157* [11]. Similarly, all referred isolates where STEC testing has been requested are tested using the same in-house PCR assays and all PCR-positive isolates are sent for sequencing. Microbiological results are stored in an in-house database, the Gastro Data Warehouse (GDW).

### STEC surveillance

In England, National Enhanced Surveillance System for STEC (NESSS) amalgamates clinical data, epidemiological data and microbiological data for all STEC cases into one centralized database [3]. The STEC enhanced surveillance questionnaires (ESQs) collect a range of information, including patient demographics, risk status, clinical conditions, food and travel histories and environmental exposures. The Public Health Operational Guidance for STEC recommends that STEC ESQs be conducted for all STEC cases, although cases infected with STEC that have the virulence profile *stx2/eae*, associated with more severe clinical outcomes and HUS, are prioritized [Shiga toxin-producing *Escherichia coli*: public health management – GOV.UK (www.gov.uk)].

Epidemiological data of STEC belonging to CC17 were extracted from NESSS, and where available, ESQ data such as information on clinical presentation, food and animal exposures and associated travel were analysed. STEC ESQ data were linked to whole-genome sequencing (WGS) data, including *stx* subtyping, virulence profiling and antimicrobial resistance (AMR) profiling, to understand the epidemiology and distribution of STEC CC17 in England. In this study, NESSS data were only available for cases from England and not for cases from Wales. Where NESSS cases were concluded to be lost to follow-up (i.e. cases were not followed up further and further epidemiological data were not collected), travel data were also obtained from microbiological results stored in GDW.

### DNA extraction, WGS and post-sequencing data processing

Genomic DNA from all isolates was extracted using the QIAsymphony (Qiagen). The sequence library was prepared using the Nextera XT kit and sequenced on the Illumina HiSeq 2500 and NextSeq 1000 platforms (100 bp paired-end reads). Generated FASTQ reads were processed using Trimmomatic v0.27 to remove bases with a PHRED score of <30 from the leading and trailing ends, with reads that were <50 bp after quality trimming being discarded.

#### Serotyping, *stx* subtyping and multi-locus sequence typing (MLST)

Post WGS, isolates were processed through an in-house bioinformatics pipeline to determine serotype and *stx* subtype via GeneFinder (https://github.com/phe-bioinformatics/gene_finder) [12]. GeneFinder was used to map FASTQ reads to the genes in the SerotypeFinder reference database using Bowtie 2 [13], and the best match to each of the O and H antigen groups were reported with quality metrics including coverage, depth, mixture and sequence homology [14]. For quality, only *in silico* predictions of serotype that matched a gene determinant with >80% nucleotide identity and >80% length were accepted. *Stx* subtyping was performed by mapping reads to variants in the *stx* variant database [15]. MLST was performed using Metric Orientated Sequence Typer (https://github.com/phe-bioinformatics/MOST) [16].

#### Virulence and AMR profiling

Virulence profiling was performed via GeneFinder (https://github.com/phe-bioinformatics/gene_finder) using the UKHSA in-house virulence database and supplemented with variants of putative genes in *E. coli* from the Centre for Genomic Epidemiology database (v2023-07-14) (https://bitbucket.org/genomicepidemiology/virulencefinder_db/src/master/virulence_ecoli.fsa). Genes were confirmed to be present if the coverage and homology were over 85% compared to the reference gene. AMR profiling was performed, and the presence of AMR genes was determined using GeneFinder (https://github.com/phe-bioinformatics/gene_finder) as well as the UKHSA in-house database. AMR profiles are displayed using Upset R (http://gehlenborglab.org/research/projects/upsetr/).

### Phylogenetic analysis

SnapperDB v0.2.8 is the UKHSA in-house database, where variant data derived from genomic DNA sequencing are stored, relative to an appropriate reference for *E. coli* CCs and other GI pathogens, to facilitate reproducible analyses of bacterial populations [17]. SnapperDB v0.2.8 was employed to generate a whole-genome alignment of isolates belonging to CC17. Gubbins v2.0.0 was used on all isolates within the alignment to identify recombinant regions that were subsequently masked after SnapperDB v0.2.8 was used again to re-extract the variants belonging to CC17 [17,18]. This generated a secondary alignment of given variant positions belonging to a minimum of 80% of the strains in the alignment. This alignment was analysed using IQTree v2.0.4, where a maximum-likelihood phylogeny was produced using the best-fit model and then visualized in ITOL v6 [19,20]. The reference genome used was AP010958.1 *Escherichia coli* O103:H2 str. 12009.

#### Single nucleotide polymorphism (SNP) cluster identification

Hierarchical single linkage clustering of pairwise Single Nucleotide Polymorphism (SNP) distances can be used to facilitate real-time detection of SNP clusters. Pairwise clustering is performed at seven descending set thresholds of SNP distance: 250, 100, 50, 25, 10, 5 and 0 SNPs. In this study, SNP clusters were defined as three or more isolates with 0 to 5 SNP differences (i.e. within the 0 SNP or 5 SNP threshold). Isolates that belong to the same SNP cluster are likely to be epidemiologically linked and/or share a common exposure.

## Results

### Overview of CC17 in England and Wales

**Fig. 1.**
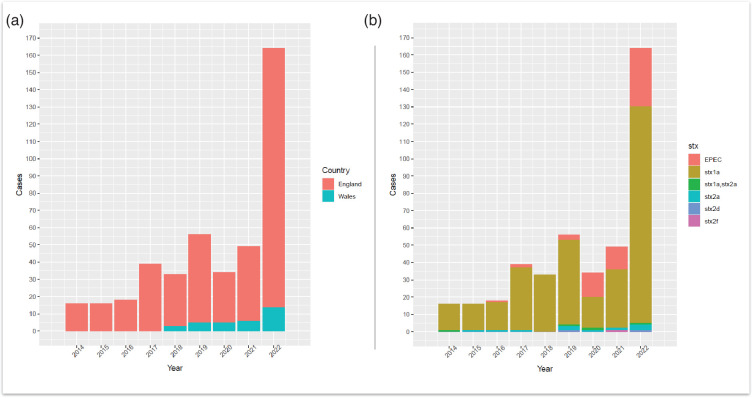
(a) Annual number of cases belonging to CC17 reported to UKHSA from 2014 to 2022, originating from England and Wales (*n*=425). (b) Cases per year of *E. coli* belonging to CC17 (*n*=425) with *stx* subtype breakdown are also represented as the different stacks for each bar. EPEC, enteropathogenic *E. coli*

**Fig. 2.**
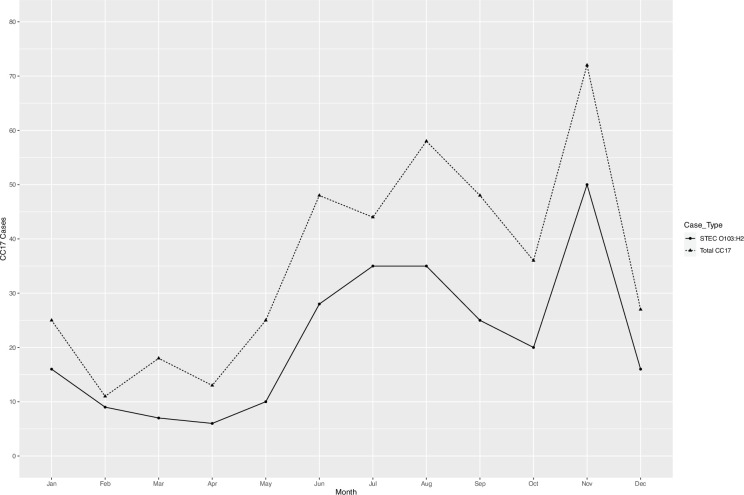
The number of STEC O103:H2 cases (*n*=257 cases) depicted by solid black line, and total CC17 cases (*n*=425) depicted by dashed line, by months in England and Wales.

There were 471 human isolates belonging to CC17 notified between January 2014 and December 2022, originating from 420 patients (Table S1, available in the online Supplementary Material). Thirty-two patients had multiple isolates of the same *E. coli* strain (i.e. defined as same *stx* and SNP profile), while five patients had multiple isolates with different strains of * E. coli* belonging to CC17 (i.e. defined as differing *stx* and/or SNP profiles). This produced an overall total of 425 unique *E. coli* strains belonging to CC17, which subsequently underwent further analysis. Overall, diagnoses of CC17 infection increased each year, from 16 total cases in 2014 to 164 cases in 2022 (Fig. 1a). The Shiga toxin subtype *stx1a* was the predominant *stx* subtype in all reported CC17 cases each year (Fig. 1b). Seasonal variation in the number of reported STEC O103:H2 and total CC17 cases observed an early summer/late autumn peak from June to October and a secondary peak in November (Fig. 2).

### Epidemiology of STEC belonging to CC17

**Fig. 3.**
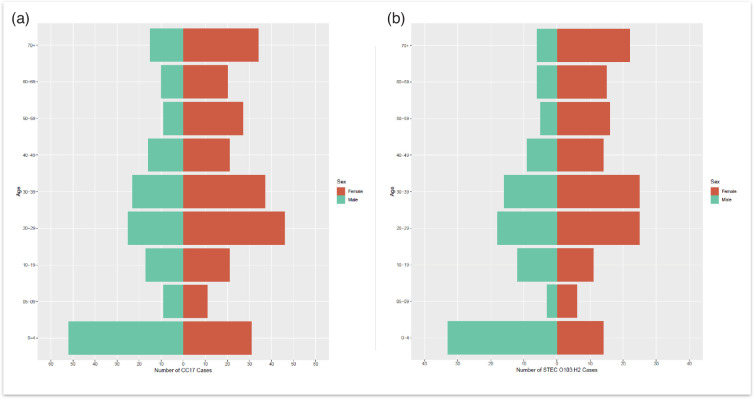
(a) Age–sex distribution of total CC17 cases reported to UKHSA [isolates originating from England and Wales (*n*=424)], where date of birth, sample receipt date and sex were available. (b) Age–sex distribution of STEC O103:H2 cases in England and Wales, where date of birth, sample receipt date and sex were available (*n*=256). Red indicates female data, and green indicates male data.

The age–sex distribution of all CC17 cases included in this study, where age and sex data were available (*n*=424), indicated that the majority of the cases were female (*n*=248/424, 58.5%) compared to male cases (*n*=176/424, 41.5%) (Fig. 3). The median age for females was 32 years (interquartile range (IQR): 19–56), while the median age for males was 24 years (IQR: 4–46). Additionally, the highest proportion of CC17 cases belonged to the 0–4 age group for males and females combined (*n*=83/424, 19.6%), as well as for males only (*n*=52/424, 12.3%). The highest proportion of CC17 cases for females belonged to the 20–29 age group (*n*=46/424, 10.8%). In the 0–4 age group, the majority of cases were male (*n*=52/424, 12.3%) compared to females (*n*=31/424, 7.3%).

CC17 cases were detected in Wales and all regions of England, with the South of England having the highest frequency (44.7%), followed by London (15.1%) and the North of England (14.8%). For CC17 STEC, the South East of England had the highest frequency of cases (46.0%), followed by London (12.8%), while the lowest frequency was observed in Yorkshire and Humber (3.7%) and the North East of England (2.7%) (Table 1).

**Table 1.** Geographical distribution of CC17 cases by region in England and Wales (*n*=425)

Region	Cases	%
London	64	15.1
North of England	63	14.8
South of England	190	44.7
East of England	25	5.9
Midlands	51	12.0
Wales	32	7.5
**Total**	**425**	**100**

Foreign travel outside the UK in the 7 days prior to onset of symptoms was reported for 25.2 % of cases, of which 36.4% were thought to be travel-related/travel-acquired. The top travel destinations for all CC17 cases were Mexico (6.1%), Egypt (3.5%) and the Dominican Republic (2.1%). The highest proportion of travellers had the serotype profile O103:H2 (48.6%), O123:H2 (20.6%) and O151:H2 (8.4%).

Clinical outcome data retrieved from NESSS were available for 46.4% of STEC CC17 cases in England. The most reported symptoms were diarrhoea (92.1%), abdominal pain (72.4%) and blood in stool (55.3%), while nausea (40.8%), fever (26.3%) and vomiting (22.4%) were also reported. In total, 20.4% of STEC cases were hospitalized and 1.3% developed HUS (Table 2). No fatalities were reported.

**Table 2.** Clinical presentation of CC17 STEC cases from England (*n*=153/330) based on available clinical outcome data retrieved from NESSS

Clinical presentation	Cases	%
Diarrhoea	140	92.1
Abdominal pain	110	72.4
Blood in stool	84	55.3
Nausea	62	40.8
Vomiting	34	22.4
Fever	40	26.3
Asymptomatic	1	0.7
**HUS**	2	1.3
Died	0	0.0
Admitted to hospital	31	20.4

### Population structure of CC17 in England and Wales

**Fig. 4.**
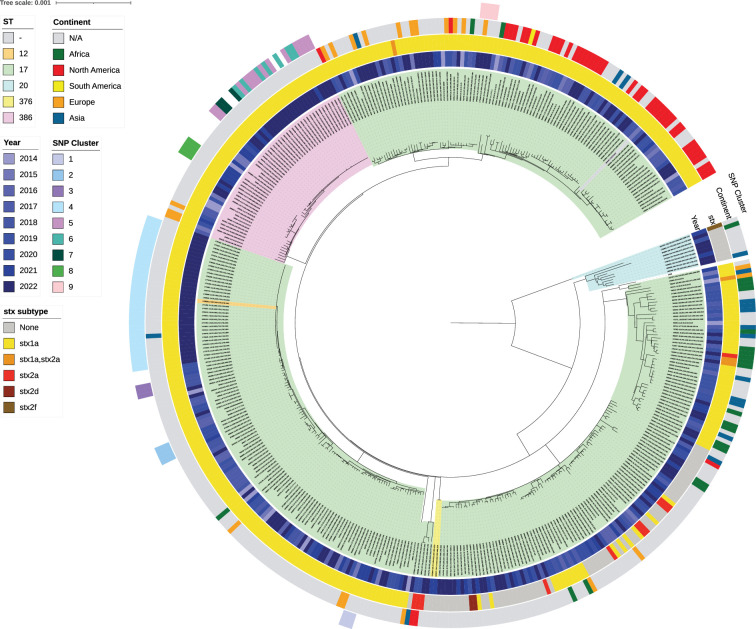
Population structure of CC17 in England and Wales (*n*=420) (rooted at midpoint) curated in IQTree2 through an alignment of variant positions against the reference genome AP010958.1 *Escherichia coli* O103:H2 str. 12009 and visualized in ITOL v6. The ST of isolates is indicated based on the coloured ranges highlighting the isolate labels and clade. In order from inwards to outwards, the year isolates were received is indicated by the first inner colour strip (blue) after the ST labels, while coloured strips designating the *stx* subtype and continent travelled follow the year. The outer coloured strip ring denotes the nine different SNP clusters identified and grouped according to SNP address (within five SNPs) and SNP distance analysis.

The five most common serotypes were O103:H2 (64.5%), O123:H2 (11.1%), O151:H2 (6.6%), O71:H2 (3.3%) and O4:H2 (2.6%). Five sequence types (STs) were identified in CC17 (ST12, ST17, ST20, ST376 and ST386), of which the majority were ST17 (83.5%) (Figs 4 and S1). In CC17, most isolates were STEC (82.5%), while 15.8% were enteropathogenic *E. coli* (EPEC). The most common virulence profile was *stx1a/eae* (78.6%), while the least common virulence profiles were *stx2f/eae* (0.2%) and *stx2d/eae* (0.5%). The presence of *eae* was confirmed in 97.4% of isolates, while *espA*, *espB*, *espF* and *tir* were detected in 96.5% isolates, confirming the presence of the LEE (Fig. 4). For virulence genes associated with the pO157 plasmid, *toxB* was detected in 27.4% isolates, while *ehxA* was detected in 92.2% isolates (Fig. S1).

### Outbreaks of CC17 in England and Wales

Nine SNP clusters were detected within CC17, where all isolates were STEC O103:H2 and had the *stx* subtype *stx1a* (Table 3 and Fig. 4). The median number of cases identified in each cluster was 4. Three clusters showed evidence of familial transmission, one cluster was geographically linked and four clusters were considered geographically dispersed. Six clusters (66.7%) were temporally related, with the duration of time between the first and last case ranging from 4 to 66 days. The two largest SNP clusters were cluster 4 (*n*=34) and cluster 5 (*n*=13) (Table 3). Cluster 4 was thought to be associated with a foodborne vehicle, but the potential source of infection was unable to be identified, while the source of infection for cluster 5 was attributed to unpasteurized soft cheese (Heinsbroek *et al*. 2024, in press, personal communication).

**Table 3.** SNP clusters of CC17 STEC cases isolated in England and Wales, identified through the generation of single linkage SNP addresses. Columns indicate the detected SNP clusters, the country/countries in which the clusters originated, the serotype and STs of each cluster, the number of cases in each cluster (*n*) and the duration of days between the first and last case of each cluster, based on the receipt date, as this was consistently reported for all cases. Asterisks (*) identify the clusters where epidemiological data indicate that familial transmission (household cases) is likely

SNP cluster	Country	Serotype	ST	Cases (*n*)	Duration between first and last case (days)
1*	Wales	O103:H2	17	3	7
2	England	O103:H2	17	4	343
3	England	O103:H2	17	3	18
4*	England/Wales	O103:H2	17	34	41
5	England/Wales	O103:H2	386	13	342/43 (excluding historical case)
6	England/Wales	O103:H2	386	7	377
7	England	O103:H2	386	3	66
8*	England	O103:H2	386	4	13
9	England	O103:H2	17	4	4

### Genome-derived AMR profiles

**Fig. 5.**
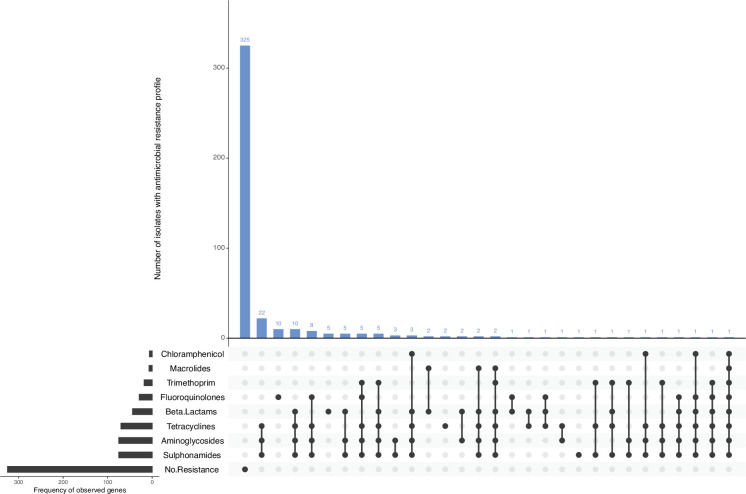
AMR profiles of CC17 isolates (*n*=425). *In silico* detection of AMR genes was performed using GeneFinder and the UKHSA AMR gene database. AMR profiles are displayed using Upset in R.

Of all CC17 isolates profiled *in silico*, 325/425 (76.5 %) were not observed to harbour any AMR determinants in the reference database, and complete susceptibility to all eight antimicrobial classes included in this analysis was inferred (Fig. 5). There were 100/425 (23.5 %) isolates detected to harbour AMR determinants conferring resistance to at least one antimicrobial class (Fig. 5).

Of 425 isolates, 44 (10.4 %) harboured AMR determinants known to confer resistance to the β-lactams, mostly *bla_TEM-1_* (*n*=29/425, 6.8%). There were 75/425 (17.6 %) isolates harbouring AMR determinants conferring resistance to aminoglycosides. The most common AMR profile conferring aminoglycoside resistance was *strA*,*strB* (*n*=65/425, 15.3%). AMR determinants known to confer reduced susceptibility or resistance to fluoroquinolones were present in 29/425 (6.8 %) isolates, either mutations in *gyrA* and *parC* genes of the quinolone-resistance determining region and/or harbouring plasmid-mediated quinolone resistance determinants. There were 7/425 (1.6 %) isolates with AMR determinants associated with macrolide resistance. There were 18/425 (4.2 %) isolates with *dfrA* genes conferring resistance towards trimethoprim, of which *dfrA-1* was the most prevalent profile (*n*=8/425, 1.9%). There were 70/425 (16.5 %) isolates detected to harbour AMR determinants conferring resistance to tetracyclines, where variants of the *tet(A*) gene were detected in 67/425 (15.8 %) isolates. There were 75/425 (17.6 %) isolates harbouring *sul* genes conferring resistance to sulphonamides – *sul-1* (*n*=6/425, 1.4%); *sul-2* (*n*=59/425, 13.9%); and *sul-3* (*n*=2/425, 0.5%). Eight isolates carried a combination of two various *sul* genes, with the profiles *sul-1,sul-2* (*n*=7/425, 1.6%) and *sul-2,sul-3* (*n*=1/425, 0.2%), respectively. There were 6/425(1.4 %) isolates harbouring AMR determinants conferring resistance to chloramphenicol. AMR determinants include *cml-1* (*n*=1/425, 0.2%); *floR* (*n*=4/425, 0.9%); and one isolate harboured the combination profile *cml-1,floR* (*n*=1/425, 0.2%).

There were 72/425 (16.9 %) isolates that harboured AMR determinants conferring resistance against three or more antimicrobial classes and were categorized as multi-drug resistant (MDR). Of these 72 MDR isolates, 24/72 (33.3 %) were associated with foreign travel, with the most common travel destinations being Mexico (*n*=5), the Dominican Republic (*n*=5) and Egypt (*n*=4). The most common resistance profile was AMR determinants associated with tetracyclines, aminoglycosides and sulphonamides resistance (*n*=22/425, 5.2%) (Fig. 5).

## Discussion

In England and Wales, the number of cases associated with CC17 has increased between 2014 and 2022, with the number of cases in 2022 being more than ten times higher than the number of cases in 2014. The increase in reported cases across England and Wales is likely due to changes in diagnostic procedures for the detection of GI pathogens, primarily the increasing number of frontline diagnostic laboratories that have implemented PCR methodologies as a part of their routine diagnostic processing of GI specimens [2,21, 22]. The adoption of chromogenic selective agar for STEC at both frontline diagnostic laboratories and within national reference laboratories such as GBRU has further facilitated the culture and isolation of non-O157 STEC serogroups [11]. The use of chromogenic selective agar within frontline and reference laboratories improves the detection capabilities and public health surveillance for non-O157 STEC, including STEC O103:H2. Reported cases of CC17 were lower in 2020 and 2021, compared to the overall year-on-year increase in notification of CC17 cases. It is likely that the emergence of SARS-CoV-2 (COVID-19) in the UK in early 2020 and the subsequent government-imposed lockdowns, restrictions on socializing, domestic and international travel and limited access to non-essential healthcare reduced the number of notifications of CC17 STEC infections for public health surveillance. In line with other GI pathogens, there was a sharp increase in notifications of STEC O103:H2 once lockdown restrictions were relaxed [23].

The increase in case numbers during the summer months reflects typical seasonality patterns for STEC [3,21, 22]. Ruminants are the primary reservoir for STEC, and during early spring to late autumn, cattle and sheep are more prone to graze outside, increasing the risk of exposure to direct contact with ruminants and/or their contaminated environment [24]. Furthermore, better weather conditions during the summer months increase the likelihood of visitations to rural areas such as petting farms, including an increase in social gatherings and inclinations, such as the consumption of salads or barbequed meat, which may be contaminated or undercooked, increasing the risk of exposure to infection [25].

Previous studies have found that the highest rates of infection were reported in rural areas associated with an increased likelihood of environmental exposure to ruminants, specifically in the Northern and Western regions of England [3,26, 27]. The higher rate of CC17 STEC cases in the South of England and London was likely due to a higher proportion of frontline diagnostic laboratories having implemented PCR in these regions at the time of the study [2,21, 22].

The higher proportion of female cases in this study has been described previously and is associated with females having a higher risk of exposure to STEC as primary food handlers, involvement in childcare and variation in food consumption [21]. High case incidences in children can be attributed to children being more likely to present to primary healthcare, although it is also possible that children are more susceptible to STEC infection due to a less well-developed immune system. The higher proportion of males in the 0–4 age group, in contrast to the overall male:female ratio, may support the hypothesis that the overall higher rate in females is due to the involvement of adult females in childcare and/or food preparation.

Clinical presentation of STEC O103:H2 was similar to STEC O157:H7 with respect to diarrhoea, bloody diarrhoea and abdominal pain, while the risk of developing HUS following STEC O103:H2 infection was much lower, with only two cases in this study reporting progression to HUS [3]. The majority of isolates in O103:H2 and CC17 had the *stx1a/eae* virulence profile, and the association between the *stx* subtype *stx1a* and bloody diarrhoea, including subsequent hospitalization, has been described in previous studies [6,28]. However, the association with the *stx1a* virulence profile and HUS is less common compared to other *stx* subtypes, such as *stx2a*. Half of the isolates positive for *stx2a* were associated with travel to North America, where the O103 serogroup is highly prevalent, while no travel was associated with the remaining isolates [29]. These data suggest that there may have been an acquisition of a *stx2a* encoding bacteriophage in specific domestic clades, contributing towards more severe clinical outcomes and HUS. The association of a few isolates with foreign travel may also possibly suggest that the *stx2a* encoding bacteriophages were imported from other countries where that specific virulence profile is more prominent. However, the lack of comprehensive travel data for all isolates in this study makes it difficult to establish the true numbers of cases presenting with CC17 infection associated with foreign travel, and the true incidence of travel-associated infections is likely under-represented in this study.

While CC11 and CC29 are almost exclusively comprised of STEC O157:H7 and STEC O26:H11, respectively [30], CC17 is composed of a multitude of serotypes, of which STEC O103:H2 is the most prominent serotype within this CC in England and Wales. A cattle survey conducted from 2014 to 2015 identified STEC O103 as the most commonly detected serogroup, surpassing STEC O26 in cattle herds and pats compared to a previous survey from 2002 to 2004, indicating a potential genuine increase in STEC O103 herd carriage within Scotland [31,32]. Although the lack of surveillance studies on STEC O103 in ruminants makes it difficult to establish the true prevalence of the STEC O103 serogroup within the ruminant population, the high prevalence of the STEC O103 serogroup in the ruminant population in the UK is corroborated by studies in other countries, where STEC O103 was the most common non-O157 serogroup present in cattle in New Zealand, Ireland, the United States, Canada and Australia [33,39].

The majority of isolates belonging to CC17 did not harbour any AMR determinants, and complete susceptibility to all eight antimicrobial classes included in this analysis was predicted based on genome-derived *in silico* AMR profiles. Previous studies highlighted that resistance to tetracyclines, aminoglycosides and sulphonamides is frequent in non-O157 STEC isolates, and these antimicrobials account for most antimicrobials sold for veterinary use [40]. Historical antimicrobial usage in animal husbandry before the implementation of agricultural regulations may have driven the acquisition of AMR determinants observed in a proportion of STEC (and CC17) in the UK.

With the exception of two household clusters, the majority of outbreaks were small, temporally related, geographically dispersed across various regions in England and Wales, and likely to be associated with foodborne transmission. However, the contaminated food vehicle was confirmed in only one of the outbreaks and was identified as unpasteurized cheese. The Advisory Committee on the Microbiological Safety of Food reported an increase in the number of raw drinking milk (RDM) producers and RDM-related outbreaks in the UK since 2015 [41,42], while outbreaks due to the consumption of unpasteurized cheeses have been reported elsewhere in Canada and the USA [43,45]. Previous outbreaks of O103:H2 have been associated with foodborne transmission involving contaminated beef and sprouts in the USA, contaminated unpasteurized milk in Germany and contaminated raw minced celery in pre-packed sandwiches in Canada [46,49].

Public health follow-up and administration of an ESQ is prioritized for higher risk STEC strains such as STEC O26 and strains causing HUS. Most isolates in this study had the virulence profile *stx1a/eae* and are considered lower risk STEC strains, so ESQs were not routinely administered, leading to a lack of epidemiological data, including information on clinical outcomes and food or animal exposures. Prioritization of cases with more severe clinical outcomes may also lead to bias in the available data of non-O157 STEC serogroups and falsely inflate rates of HUS. The analysis of available surveillance data to assess the incidence and prevalence of CC17 in England and Wales is challenging due to inconsistencies in the referral and reporting algorithms for non-O157 STEC across the UK. The lack of comprehensive microbiological and/or epidemiological surveillance systems has a severe impact on assessing the overall burden of STEC O103:H2 and CC17 in England and Wales. Expediting PCR implementation is crucial to increase the capacity of detecting CC17 cases and enhance surveillance. Genomics is needed to inform the epidemiology of STEC CC17, and microbiological data and epidemiological data need to be analysed in conjunction to build a detailed picture of the pathogenicity and impact of CC17 STEC.

## supplementary material

10.1099/jmm.0.001928 Uncited Supplementary Material 1.

## Abbreviations

AMR: antimicrobial resistance
CC: clonal complex
CT-SMAC: cefixime tellurite sorbitol MacConkey
DEC: diarrhoeagenic *E. coli*
ESQ: enhanced surveillance questionnaire
GBRU: Gastrointestinal Bacteria Reference Unit
GDW: Gastro Data Warehouse
GI: gastrointestinal
HUS: haemolytic uraemic syndrome
LEE: locus of enterocyte effacement
MDR: multi-drug resistant
MLST: multi-locus sequence typing
NESSS: National Enhanced Surveillance System for STEC
RDM: raw drinking milk
SNP: single nucleotide polymorphism
ST: sequence type
STEC: Shiga toxin-producing *Escherichia coli*
*stx*: Shiga toxin
UKHSA: UK Health Security Agency
WGS: whole -genome sequencing
