# Supplementary material for: Genotypic analysis of Shiga toxin-producing Escherichia coli clonal complex 17 in England and Wales, 2014–2022
Source: J Med Microbiol. 2024 Nov 7;73(11):001928. doi: 10.1099/jmm.0.001928 (PMC11542628; doi:10.1099/jmm.0.001928)
Supplement: Uncited Supplementary Material 1. [file jmm-73-01928-s001.pdf]

Supplementary Figures

Supplementary Figure 1: Population structure of CC17 in England and Wales (*n*=423) (2 isolates excluded). Isolate labels are colour coded according to their respective ST. The distribution of virulence factors for each isolate in the population structure of CC17 are presented as a heatmap, using virulence genes retrieved from the UKHSA in-house virulence database and CGE database. Blue represents virulence genes which are present (where gene coverage and homology were >85%), while white represents virulence genes which are absent.

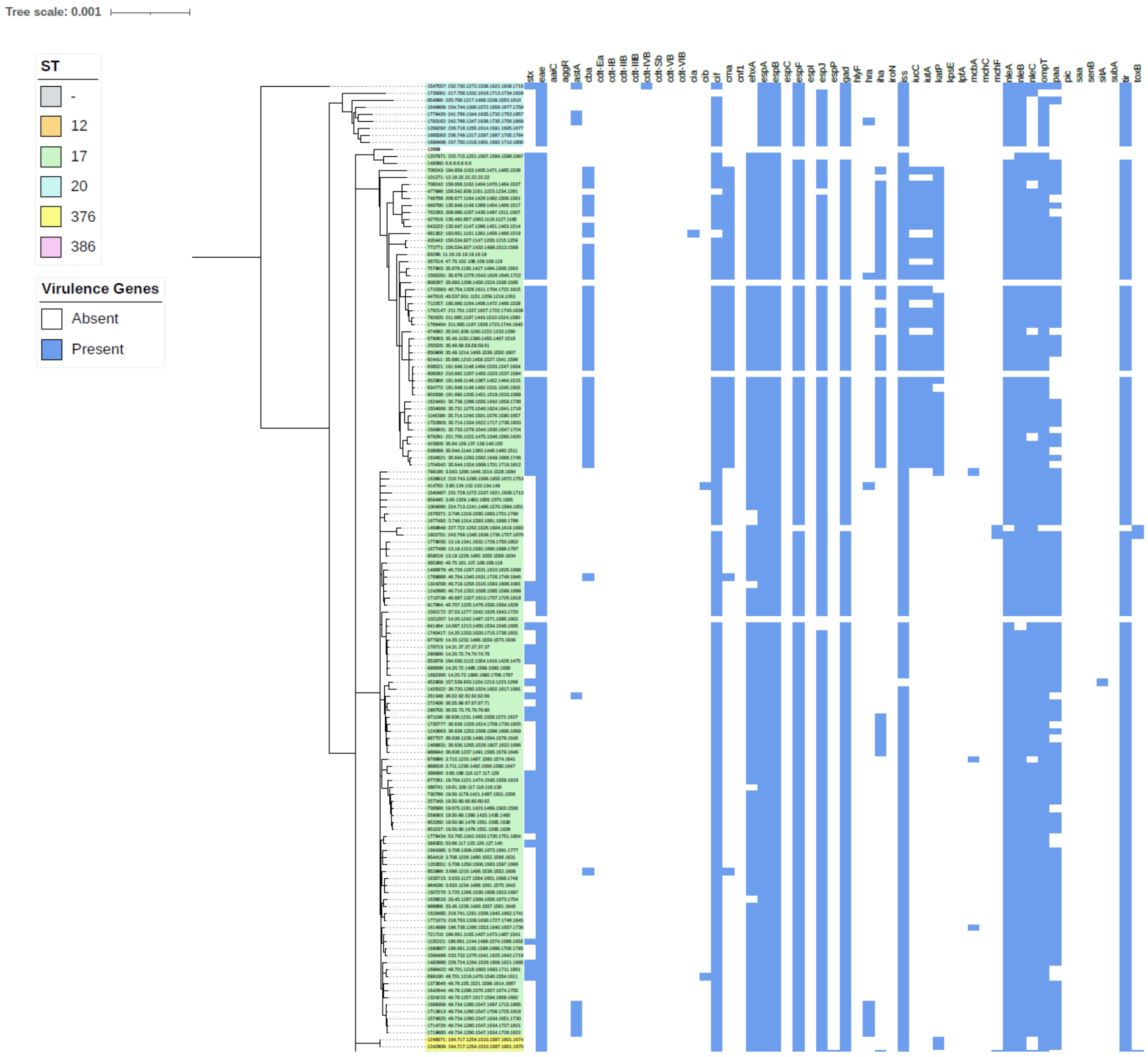

Supplementary Tables

Supplementary Table 1: Metadata and SRA accessions of isolates included in this study.

SRA Accession	Receipt date	Sex	Travel Destination (GDW)	Serotype	ST	CC	Stx Subtype
SRR3240967	2014	M	N/A	O103:H2	17	CC17	stx1a
SRR9942560	2019	F	Cuba	O103:H2	17	CC17	stx1a
SRR9942569	2019	F	Caribbean	O103:H2	17	CC17	stx1a
SRR5024462	2015	F	Mexico	O103:H2	17	CC17	stx1a
SRR4897587	2016	F	Unknown	O103:H2	17	CC17	stx1a
SRR3578621	2015	F	Mexico	O103:H2	-	CC17	stx1a
SRR7184238	2017	M	Mexico	O103:H2	17	CC17	stx1a
SRR7291942	2017	F	Mexico	O103:H2	17	CC17	stx1a
SRR5024503	2016	F	Mexico	O103:H2	17	CC17	stx1a
SRR7892569	2018	M	Dominican republic	O103:H2	17	CC17	stx1a
SRR7943932	2018	M	Unknown	O103:H2	17	CC17	stx1a
SRR7291432	2015	M	N/A	O103:H2	17	CC17	stx1a
SRR20993566	2022	M	Mexico	O103:H2	17	CC17	stx1a
SRR7407920	2017	M	Unknown	O103:H2	17	CC17	stx1a
SRR9335424	2019	M	Unknown	O103:H2	17	CC17	stx1a
SRR10874264	2019	F	Mexico	O103:H2	17	CC17	stx1a
	2022	M	Unknown	O103:H2	17	CC17	stx1a
SRR21525080	2022	M	South America	O103:H2	17	CC17	stx1a
SRR10357091	2019	F	Unknown	O103:H2	17	CC17	stx1a
SRR10858713	2019	M	Mexico	O103:H2	17	CC17	stx2a
SRR10858710	2019	M	Mexico	O103:H2	17	CC17	stx2a
SRR19925128	2022	M	Unknown	O103:H2	17	CC17	stx2a
SRR5033707	2014	F	N/A	O Unidentifiable	17	CC17	stx1a
SRR5023726	2014	F	Unknown	O Unidentifiable	17	CC17	stx1a,stx2a
SRR12175228	2020	M	Unknown	O71:H2	17	CC17	-
SRR20912088	2022	M	Unknown	O71:H2	17	CC17	-
SRR22520713	2022	F	Unknown	O71:H2	17	CC17	-
SRR7286448	2017	M	Pakistan	O71:H2	17	CC17	stx1a
	2018	M	Pakistan	O71:H2	17	CC17	stx1a
SRR8466388	2019	M	Unknown	O71:H2	17	CC17	stx1a
SRR12535025	2020	M	N/A	O123:H2	17	CC17	-
SRR13057902	2020	M	N/A	O123:H2	17	CC17	-
SRR22179367	2022	M	Unknown	O123:H2	17	CC17	-
SRR12798029	2020	M	N/A	O123:H2	17	CC17	-
SRR21404549	2022	F	Unknown	O123:H2	17	CC17	-
SRR5006434	2016	M	Unknown	O123:H2	17	CC17	stx2a
SRR3578585	2015	F	N/A	O186:H2	17	CC17	stx2a
SRR10513265	2019	F	Unknown	O123-O186:H2	17	CC17	stx1a
SRR7221260	2017	F	Unknown	O103:H2	17	CC17	stx1a
SRR7249773	2017	M	Unknown	O103:H2	17	CC17	stx1a
SRR9678943	2019	F	Unknown	O103:H2	17	CC17	stx1a
SRR7172301	2017	F	Unknown	O4:H2	17	CC17	stx1a

SRR7358292	2018	F	Egypt	O103:H2	17	CC17	stx1a
SRR8774279	2019	F	Egypt	O103:H2	17	CC17	stx1a
SRR15035460	2021	F	Unknown	O103:H2	376	CC17	-
SRR15043704	2021	M	Unknown	O103:H2	376	CC17	-
	2019	M	Mexico	O123:H2	17	CC17	stx1a
SRR18589147	2022	F	Mexico	O123:H2	17	CC17	stx1a
SRR7401643	2018	F	Unknown	O123:H2	17	CC17	stx1a
SRR8086691	2018	M	Mexico	O123:H2	17	CC17	stx1a
	2018	F	Mexico	O123:H2	17	CC17	stx1a
SRR9836904	2019	F	Mexico	O123:H2	17	CC17	stx1a
SRR11361762	2020	F	Mexico	O123:H2	17	CC17	stx1a
SRR17281177	2021	F	Unknown	O123:H2	17	CC17	stx1a
SRR19139249	2022	M	Dominican republic	O123:H2	17	CC17	stx1a
SRR19349862	2022	F	Dominican republic	O123:H2	17	CC17	stx1a
	2022	F	Dominican republic	O123:H2	17	CC17	stx1a
SRR3241862	2014	F	Mexico	O186:H2	17	CC17	stx1a
SRR7215900	2017	F	Unknown	O123:H2	17	CC17	stx1a
SRR3574288	2016	F	Mexico	O186:H2	17	CC17	stx1a
SRR7277693	2015	F	Mexico	O186:H2	17	CC17	stx1a
SRR7184347	2017	F	Unknown	O123:H2	17	CC17	stx1a
SRR7274802	2017	F	Unknown	O123:H2	17	CC17	stx1a
SRR7186942	2017	M	Mexico	O123-O186:H2	17	CC17	stx1a
	2019	F	Mexico	O123:H2	17	CC17	stx1a
SRR7367229	2018	M	Unknown	O123:H2	17	CC17	stx1a
SRR8981812	2019	M	Unknown	O71:H2	17	CC17	stx1a
SRR7439075	2018	F	N/A	O103:H2	17	CC17	stx1a
SRR12010338	2020	M	Unknown	O71:H2	17	CC17	stx1a
SRR12010388	2020	M	Unknown	O71:H2	17	CC17	stx1a
SRR4897311	2016	F	Unknown	O71:H2	17	CC17	stx1a
SRR9050436	2019	F	Egypt	O71:H2	17	CC17	stx1a
SRR10971394	2020	F	Egypt	O71:H2	17	CC17	stx1a
SRR7230251	2017	M	Unknown	O71:H2	17	CC17	stx1a
SRR8307277	2018	M	India	O111:H2	17	CC17	stx1a
SRR10419588	2019	M	India	O111:H2	17	CC17	stx1a
SRR10466979	2019	F	Unknown	O111:H2	17	CC17	stx1a
SRR10120195	2019	F	Unknown	O111:H2	17	CC17	stx1a
SRR8558528	2019	F	Pakistan	O103:H2	17	CC17	stx1a
SRR8774311	2019	F	India	O109:H2	17	CC17	stx1a
SRR8820012	2019	M	Egypt	O123:H2	17	CC17	stx1a
SRR8873615	2019	F	Unknown	O123:H2	17	CC17	-
SRR21284066	2022	F	Unknown	O123:H2	17	CC17	-
SRR13968830	2021	M	Unknown	O123:H2	17	CC17	stx1a
SRR18820643	2022	F	Unknown	O123:H2	17	CC17	-
SRR3241841	2014	M	N/A	O103:H2	17	CC17	stx1a
SRR21918434	2022	M	Morocco	O103:H2	17	CC17	stx1a
SRR3241847	2014	F	N/A	O103:H2	17	CC17	stx1a

SRR7842182	2018	F	N/A	O103:H2	17	CC17	stx1a
SRR19806054	2022	M	N/A	O103:H2	17	CC17	stx1a
SRR20273474	2022	F	Unknown	O103:H2	17	CC17	stx1a
SRR7290915	2018	F	Unknown	O103:H2	17	CC17	stx1a
SRR7416069	2018	M	Unknown	O103:H2	17	CC17	stx1a
SRR16941066	2021	M	Unknown	O103:H2	17	CC17	stx1a
SRR19926125	2022	M	N/A	O103:H2	17	CC17	stx1a
SRR17893045	2022	F	N/A	O103:H2	17	CC17	stx1a
SRR19785308	2022	M	Unknown	O103:H2	17	CC17	stx1a
SRR12062826	2020	M	Unknown	O103:H2	17	CC17	stx1a
SRR17696134	2022	F	N/A	O103:H2	17	CC17	stx1a
SRR17704100	2022	F	Unknown	O103:H2	17	CC17	stx1a
SRR19737553	2022	F	Unknown	O103:H2	17	CC17	stx1a
SRR23052070	2022	M	Unknown	O103:H2	17	CC17	stx1a
SRR3241850	2014	M	N/A	O103:H2	17	CC17	stx1a
SRR7291007	2016	M	N/A	O103:H2	17	CC17	stx1a
SRR6185886	2017	M	N/A	O unidentifiable:H2	17	CC17	stx1a
SRR10313623	2019	F	Greece	O103:H2	17	CC17	stx1a
SRR15038691	2021	F	Unknown	O103:H2	17	CC17	stx1a
SRR15842838	2021	F	Unknown	O103:H2	17	CC17	stx1a
SRR15843079	2021	M	Unknown	O unidentifiable:H2	17	CC17	stx1a
SRR17900596	2022	M	Unknown	O103:H2	17	CC17	stx1a
SRR4192137	2015	M	N/A	O103:H2	17	CC17	stx1a
SRR22432229	2022	M	N/A	O103:H2	17	CC17	stx1a
SRR5005304	2016	F	N/A	O103:H2	17	CC17	stx1a
	2019	M	Unknown	O103:H2	17	CC17	stx1a
SRR13153592	2020	F	N/A	O103:H2	17	CC17	stx1a
SRR20721008	2022	F	Unknown	O103:H2	17	CC17	stx1a
SRR21910741	2022	F	N/A	O103:H2	17	CC17	stx1a
SRR5023767	2014	F	Italy	O103:H2	17	CC17	stx1a
SRR9720385	2019	F	Unknown	O103:H2	17	CC17	stx1a
SRR15299577	2021	M	Unknown	O103:H2	17	CC17	stx1a
SRR19431957	2022	M	Unknown	O103:H2	17	CC17	stx1a
	2021	F	Unknown	O103:H2	17	CC17	stx1a
	2021	M	N/A	O103:H2	17	CC17	stx1a
	2021	M	N/A	O103:H2	17	CC17	stx1a
SRR15183681	2021	F	Unknown	O103:H2	17	CC17	stx1a
SRR7191684	2015	M	Unknown	O103:H2	17	CC17	stx1a
SRR3581323	2015	F	Unknown	O103:H2	17	CC17	stx1a
SRR15842357	2021	M	Unknown	O103:H2	17	CC17	stx1a
SRR9050431	2019	M	N/A	O103:H2	17	CC17	stx1a
SRR7223136	2017	F	N/A	O103:H2	17	CC17	stx1a
SRR7215285	2017	F	N/A	O103:H2	17	CC17	stx1a
SRR10198864	2019	F	Unknown	O103:H2	17	CC17	stx1a
SRR11361771	2020	M	Unknown	O103:H2	17	CC17	stx1a

SRR4181493	2015	F	N/A	O103:H2	17	CC17	stx1a
SRR18008301	2022	M	N/A	O103:H2	17	CC17	stx1a
SRR19737560	2022	M	Unknown	O103:H2	17	CC17	stx1a
SRR7191470	2017	F	N/A	O103:H2	17	CC17	stx1a
SRR7266778	2017	F	Unknown	O103:H2	17	CC17	stx1a
SRR7842202	2018	M	N/A	O103:H2	17	CC17	stx1a
SRR7943913	2018	F	Unknown	O103:H2	17	CC17	stx1a
SRR7184213	2017	F	Unknown	O103:H2	17	CC17	stx1a
SRR12263118	2020	M	Unknown	O103:H2	17	CC17	stx1a
SRR12492526	2020	F	Unknown	O103:H2	17	CC17	stx1a
SRR12535061	2020	F	Unknown	O103:H2	17	CC17	stx1a
SRR15038686	2021	F	Unknown	O103:H2	17	CC17	stx1a
SRR12630740	2020	M	Unknown	O103:H2	17	CC17	stx1a
SRR19333117	2022	M	Unknown	O103:H2	17	CC17	stx1a
SRR7163937	2017	F	N/A	O103:H2	17	CC17	stx1a
SRR7358006	2018	F	Unknown	O103:H2	17	CC17	stx1a
SRR7407952	2018	M	Unknown	O103:H2	17	CC17	stx1a
SRR7474034	2018	F	Unknown	O103:H2	17	CC17	stx1a
SRR10036694	2019	M	Unknown	O103:H2	17	CC17	stx1a
SRR8700959	2019	M	N/A	O103:H2	17	CC17	stx1a
SRR8820005	2019	M	Unknown	O103:H2	17	CC17	stx1a
SRR10962106	2020	F	Unknown	O103:H2	17	CC17	stx1a
SRR22469488	2022	M	Unknown	O103:H2	17	CC17	stx1a
SRR22578050	2022	F	N/A	O103:H2	17	CC17	stx1a
SRR22640879	2022	M	Unknown	O103:H2	17	CC17	stx1a
SRR22578051	2022	M	Unknown	O103:H2	17	CC17	stx1a
SRR22640892	2022	M	N/A	O103:H2	17	CC17	stx1a
SRR22432284	2022	M	N/A	O103:H2	17	CC17	stx1a
SRR22516089	2022	F	Unknown	O103:H2	17	CC17	stx1a
SRR22516081	2022	F	N/A	O103:H2	17	CC17	stx1a
SRR22640840	2022	F	Unknown	O103:H2	17	CC17	stx1a
SRR22520728	2022	F	N/A	O103:H2	17	CC17	stx1a
SRR22520715	2022	F	N/A	O103:H2	17	CC17	stx1a
SRR22469486	2022	F	Unknown	O103:H2	17	CC17	stx1a
SRR22422882	2022	F	Unknown	O103:H2	17	CC17	stx1a
SRR22520720	2022	M	Unknown	O103:H2	17	CC17	stx1a
SRR22578054	2022	M	Unknown	O103:H2	17	CC17	stx1a
SRR22516088	2022	M	N/A	O103:H2	17	CC17	stx1a
SRR22508447	2022	M	Unknown	O103:H2	17	CC17	stx1a
SRR22516079	2022	F	N/A	O103:H2	17	CC17	stx1a
SRR22516098	2022	F	N/A	O103:H2	17	CC17	stx1a
	2022	F	N/A	O103:H2	17	CC17	stx1a
	2022	F	N/A	O103:H2	12	CC17	stx1a
SRR22432233	2022	F	Unknown	O103:H2	17	CC17	stx1a
SRR22432291	2022	M	Unknown	O103:H2	17	CC17	stx1a
	2022	F	Unknown	O103:H2	17	CC17	stx1a

SRR22509187	2022	F	Unknown	O103:H2	17	CC17	stx1a
SRR22516107	2022	F	Unknown	O103:H2	17	CC17	stx1a
	2022	F	Unknown	O103:H2	17	CC17	stx1a
SRR22578061	2022	F	Unknown	O103:H2	17	CC17	stx1a
SRR22578052	2022	M	Malaysia	O103:H2	17	CC17	stx1a
SRR22650928	2022	F	N/A	O103:H2	17	CC17	stx1a
SRR22775842	2022	F	Unknown	O103:H2	17	CC17	stx1a
SRR22801724	2022	F	Unknown	O103:H2	17	CC17	stx1a
SRR22801736	2022	M	N/A	O103:H2	17	CC17	stx1a
SRR23010365	2022	M	N/A	O103:H2	17	CC17	stx1a
SRR17848304	2022	M	Unknown	O103:H2	17	CC17	stx1a
SRR21129384	2022	M	Unknown	O103:H2	17	CC17	stx1a
SRR5023707	2014	F	N/A	O Unidentifiable	17	CC17	stx1a
SRR7286563	2017	F	Unknown	O103:H2	17	CC17	stx1a
SRR7230388	2017	F	N/A	O103:H2	17	CC17	stx1a
SRR7204370	2017	F	N/A	O103:H2	17	CC17	stx1a
SRR4786263	2016	F	Unknown	O103:H2	17	CC17	stx1a
SRR7187033	2016	F	Unknown	O103:H2	17	CC17	stx1a
SRR7401609	2017	F	Unknown	O103:H2	17	CC17	stx1a
SRR11361818	2020	F	Unknown	O103:H2	17	CC17	stx1a
SRR11215152	2020	F	Unknown	O103:H2	17	CC17	stx1a
SRR22099610	2022	F	Unknown	O103:H2	17	CC17	stx1a
SRR7265738	2016	F	Unknown	O103:H2	17	CC17	stx1a
SRR7244330	2016	F	N/A	O103:H2	17	CC17	stx1a
SRR3241865	2014	F	N/A	O103:H2	17	CC17	stx1a
SRR7291000	2018	M	Unknown	O103:H2	17	CC17	stx1a
SRR7215566	2018	F	N/A	O103:H2	17	CC17	stx1a
SRR9178551	2019	M	Egypt	O103:H2	17	CC17	stx1a
SRR9586547	2019	M	Unknown	O71:H2	17	CC17	stx1a
SRR19509909	2022	F	Unknown	O177:H2	17	CC17	-
SRR10013364	2019	F	Egypt	O123:H2	17	CC17	stx1a,stx2a
SRR22418060	2022	M	Egypt	O123:H2	17	CC17	stx1a,stx2a
SRR22404917	2022	F	Egypt	O123:H2	17	CC17	stx2a
	2019	M	Unknown	O111:H2	17	CC17	stx1a
SRR22085111	2022	M	Uganda	O128ab:H2	20	CC17	-
SRR19171592	2022	M	N/A	O109:H32	17	CC17	-
SRR22432275	2022	M	Unknown	O109:H32	17	CC17	-
SRR10698122	2019	F	N/A	O128ab:H2	20	CC17	-
	2020	M	N/A	O123:H2	17	CC17	stx1a
SRR12768320	2020	F	Unknown	O103:H2	17	CC17	stx1a
SRR12827956	2020	M	Unknown	O177:H2	17	CC17	-
SRR14857279	2021	F	Unknown	O103:H2	17	CC17	stx1a
SRR15319354	2021	F	Unknown	O15:H2	20	CC17	-
SRR16077455	2021	F	Nigeria	O45:H2	17	CC17	-
SRR16277977	2021	F	N/A	O151:H2	17	CC17	stx1a
SRR17144086	2021	M	Mexico	O45:H2	17	CC17	-

SRR17269273	2021	M	N/A	O128ab:H2	20	CC17	stx2f
SRR17622297	2022	M	Unknown	O123:H2	17	CC17	-
	2022	F	N/A	O35:H2	20	CC17	-
SRR19902617	2022	F	Unknown	O151:H2	17	CC17	stx1a
SRR21143151	2022	F	Unknown	O15:H2	20	CC17	-
SRR21465923	2022	F	Turkey	O15:H2	20	CC17	-
SRR21628764	2022	F	India	O103:H2	17	CC17	-
SRR22432279	2022	F	Unknown	O103:H2	17	CC17	-
SRR22578109	2022	M	Unknown	O128ab:H2	20	CC17	-
SRR22851856	2022	F	Unknown	O10:H2	20	CC17	-
SRR22942742	2022	M	Gambia	O45:H2	17	CC17	-
SRR10056643	2019	F	N/A	O103:H2	17	CC17	stx1a
SRR19405233	2022	F	Unknown	O103:H2	17	CC17	-
SRR12625818	2020	M	Unknown	O103:H2	17	CC17	-
SRR12175201	2020	M	N/A	O103:H2	17	CC17	-
SRR10693133	2019	F	Unknown	O103:H2	17	CC17	-
	2020	F	Unknown	O103:H2	17	CC17	-
SRR14794500	2021	M	Unknown	O103:H2	17	CC17	-
SRR20273459	2022	F	Unknown	O103:H2	17	CC17	-
SRR12541344	2020	M	Unknown	O103:H2	17	CC17	-
SRR12676731	2020	F	Unknown	O103:H2	17	CC17	-
SRR16568633	2021	F	Unknown	O103:H2	17	CC17	-
SRR20911741	2022	F	Unknown	O103:H2	17	CC17	-
SRR20993560	2022	F	N/A	O103:H2	17	CC17	-
SRR7244271	2017	F	N/A	O103:H2	17	CC17	stx1a
SRR7187869	2017	F	N/A	O103:H2	17	CC17	-
SRR12676720	2020	M	Unknown	O116:H2	17	CC17	-
SRR19524968	2022	F	Unknown	O116:H2	17	CC17	-
SRR8547514	2019	M	Unknown	O103:H2	17	CC17	stx1a
SRR10605718	2019	F	Unknown	O123:H2	17	CC17	stx1a
SRR5024191	2016	F	India	O186:H2	17	CC17	stx1a
SRR7184242	2018	M	N/A	O123:H2	17	CC17	stx1a
SRR8201575	2018	F	Unknown	O123:H2	17	CC17	stx1a
SRR19347600	2022	M	N/A	O123:H2	17	CC17	stx1a
SRR21607685	2022	F	Turkey	O123:H2	17	CC17	stx1a
SRR9306492	2019	F	North Africa	O123:H2	17	CC17	stx1a
SRR17704182	2022	M	N/A	O123:H2	17	CC17	stx1a
SRR10212137	2019	F	Unknown	O123:H2	17	CC17	stx1a
SRR10314877	2019	F	Unknown	O123:H2	17	CC17	stx1a
SRR14087961	2021	M	Unknown	O123:H2	17	CC17	stx1a
SRR22324857	2022	F	Unknown	O123:H2	17	CC17	stx1a
	2021	F	Unknown	O123:H2	17	CC17	stx1a
	2022	F	Egypt	O123:H2	17	CC17	stx1a
SRR19139396	2022	F	Egypt	O123:H2	17	CC17	stx1a
SRR7221236	2017	M	Unknown	O103:H2	17	CC17	stx1a
SRR7274826	2016	M	N/A	O4:H2	17	CC17	stx1a

SRR4897097	2016	M	Unknown	O4:H2	17	CC17	-
SRR5023945	2016	F	Unknown	O4:H2	17	CC17	stx1a
	2020	M	Unknown	O4:H2	17	CC17	stx2a
	2020	F	Unknown	O4:H2	17	CC17	-
SRR12683915	2020	F	Unknown	O4:H2	17	CC17	-
SRR15035482	2021	F	Unknown	O4:H2	17	CC17	-
	2021	F	Unknown	O4:H2	17	CC17	-
SRR21986796	2022	F	Unknown	O4:H2	17	CC17	-
SRR15842587	2021	M	Unknown	O4:H2	17	CC17	-
SRR17636716	2022	F	Unknown	O49:H2	17	CC17	-
SRR18401507	2022	F	Unknown	O151:H2	17	CC17	stx1a
SRR19806042	2022	M	Costa rica	O151:H2	17	CC17	stx1a
SRR4787864	2016	F	Mexico	O118:H2	17	CC17	stx1a
SRR7215528	2017	M	Vietnam	OgC4-O118-O151	17	CC17	stx1a
SRR7291628	2018	F	Cambodia	O151:H2	17	CC17	stx1a
SRR7850050	2018	M	Dominican republic	O151:H2	17	CC17	stx1a
SRR10350830	2019	F	Unknown	O151:H2	17	CC17	stx1a
SRR10553795	2019	F	Dominican republic	O151:H2	17	CC17	stx1a
SRR7943929	2018	M	N/A	O151:H2	17	CC17	stx1a
SRR8097434	2018	F	Cuba	O151:H2	17	CC17	stx1a
SRR8194846	2018	F	Cuba	O151:H2	17	CC17	stx1a
SRR19324830	2022	F	Unknown	O151:H2	17	CC17	stx1a
SRR20273468	2022	M	Unknown	O151:H2	17	CC17	stx1a
SRR20428644	2022	M	Unknown	O151:H2	17	CC17	stx1a
SRR21504808	2022	F	Carribean and Mexico	O151:H2	17	CC17	stx1a
SRR7249825	2016	F	Costa rica	O118:H2	17	CC17	stx1a
SRR9083705	2019	F	Unknown	O151:H2	17	CC17	stx1a
SRR21466075	2022	M	Jamaica	O103:H2	17	CC17	stx1a
SRR22085047	2022	F	Mexico	O151:H2	17	CC17	stx1a
SRR22085218	2022	F	Unknown	O151:H2	17	CC17	stx1a
SRR7407922	2018	F	Dominican republic	O103:H2	17	CC17	stx1a
SRR4897266	2016	M	Unknown	O103:H2	17	CC17	stx1a
SRR7277768	2018	F	Unknown	O103:H2	386	CC17	stx1a
SRR15460217	2021	M	Unknown	O103:H2	386	CC17	stx1a
SRR12625831	2020	M	Unknown	O103:H2	386	CC17	stx1a
SRR16566158	2021	M	Unknown	O103:H2	386	CC17	stx1a
SRR16920103	2021	F	N/A	O103:H2	386	CC17	stx1a
SRR15842482	2021	M	Unknown	O103:H2	386	CC17	stx1a
SRR19638714	2022	F	Unknown	O103:H2	386	CC17	stx1a
SRR19854685	2022	F	Unknown	O103:H2	386	CC17	stx1a
SRR19902586	2022	F	N/A	O103:H2	386	CC17	stx1a
SRR19868596	2022	M	Unknown	O103:H2	386	CC17	stx1a
	2022	M	Unknown	O103:H2	386	CC17	stx1a
SRR20273457	2022	M	N/A	O103:H2	386	CC17	stx1a
	2022	M	Unknown	O103:H2	386	CC17	stx1a

SRR20230863	2022	F	Unknown	O103:H2	386	CC17	stx1a
SRR20230833	2022	M	Unknown	O103:H2	386	CC17	stx1a
SRR20230865	2022	F	Unknown	O103:H2	386	CC17	stx1a
SRR20331153	2022	F	Unknown	O103:H2	386	CC17	stx1a
SRR20673751	2022	F	Unknown	O103:H2	386	CC17	stx1a
SRR16966780	2021	F	Unknown	O103:H2	386	CC17	stx1a
SRR21413069	2022	F	Unknown	O103:H2	386	CC17	stx1a
SRR21465907	2022	M	N/A	O103:H2	386	CC17	stx1a
SRR21520532	2022	F	N/A	O103:H2	386	CC17	stx1a
SRR21910587	2022	F	Unknown	O103:H2	386	CC17	stx1a
	2022	F	Unknown	O103:H2	386	CC17	stx1a
SRR22340599	2022	M	Unknown	O103:H2	386	CC17	stx1a
SRR17144057	2021	F	Unknown	O103:H2	386	CC17	stx1a
SRR17874776	2022	F	Unknown	O103:H2	386	CC17	stx1a
SRR18008279	2022	M	Unknown	O103:H2	386	CC17	stx1a
SRR21918415	2022	F	Unknown	O103:H2	386	CC17	stx1a
SRR16892798	2021	F	Unknown	O103:H2	386	CC17	stx1a
SRR21413073	2022	F	Unknown	O103:H2	386	CC17	stx1a
SRR21918455	2022	M	Unknown	O103:H2	386	CC17	stx1a
SRR3241110	2014	F	Unknown	O103:H2	386	CC17	stx1a
SRR17055443	2021	F	Unknown	O103:H2	386	CC17	stx1a
SRR17099027	2021	F	Unknown	O103:H2	386	CC17	stx1a
SRR17099024	2021	M	Unknown	O103:H2	386	CC17	stx1a
SRR17205289	2021	M	Unknown	O103:H2	386	CC17	stx1a
SRR3241845	2014	M	N/A	O103:H2	386	CC17	stx1a
SRR3241981	2014	F	Unknown	O103:H2	386	CC17	stx1a
SRR3578924	2015	M	Unknown	O103:H2	386	CC17	stx1a
SRR3581364	2015	F	N/A	O103:H2	386	CC17	stx1a
	2019		Unknown	O103:H2	386	CC17	stx1a
SRR15319334	2021	M	Unknown	O103:H2	386	CC17	stx1a
	2022	M	N/A	O103:H2	386	CC17	stx1a
SRR19973577	2022	M	Unknown	O103:H2	386	CC17	stx1a
SRR22324931	2022	F	Unknown	O103:H2	386	CC17	stx1a
SRR3581457	2015	F	N/A	O103:H2	386	CC17	stx1a
SRR3578637	2015	F	Unknown	O103:H2	386	CC17	stx1a
SRR7407925	2017	M	Unknown	O103:H2	386	CC17	stx1a
SRR14407959	2021	F	N/A	O103:H2	386	CC17	stx1a
SRR10005844	2019	F	Montenegro	O103:H2	386	CC17	stx1a
SRR16077438	2021	M	Unknown	O103:H2	386	CC17	stx1a
SRR7285487	2017	F	N/A	O103:H2	386	CC17	stx1a
SRR10013360	2019	F	Unknown	O103:H2	386	CC17	stx1a
SRR7236600	2017	F	Unknown	O103:H2	386	CC17	stx1a
SRR7286364	2017	F	Egypt	O123-O186:H2	17	CC17	stx1a
	2022	M	Unknown	O123:H2	17	CC17	stx1a
SRR21910383	2022	F	Unknown	O unidentifiable:H2	17	CC17	stx2a

SRR11479169	2020	F	Unknown	O unidentifiable:H2	17	CC17	-
	2021	F	Unknown	O unidentifiable:H2	17	CC17	stx2a
SRR15512801	2021	M	Unknown	O unidentifiable:H2	17	CC17	stx1a
SRR16566153	2021	M	N/A	O unidentifiable:H2	17	CC17	-
SRR6185865	2017	M	N/A	O unidentifiable:H2	17	CC17	-
	2022	M	Unknown	O unidentifiable:H2	17	CC17	-
SRR7367206	2017	F	Pakistan	O123-O186:H2	17	CC17	stx1a
SRR11361737	2019	F	Unknown	O151:H2	17	CC17	stx2d
SRR21465928	2022	M	Unknown	O151:H2	17	CC17	stx2d
SRR18008295	2022	F	Unknown	O151:H2	17	CC17	-
SRR21910344	2022	F	Unknown	O151:H2	17	CC17	-
SRR21918459	2022	F	N/A	O151:H2	17	CC17	-
SRR21504771	2022	F	Unknown	O151:H2	17	CC17	-
SRR21918523	2022	M	N/A	O151:H2	17	CC17	-
SRR15842530	2021	F	Unknown	O151:H2	17	CC17	-
SRR15512836	2021	M	Unknown	O151:H2	17	CC17	-
SRR19601376	2022	F	Unknown	O151:H2	17	CC17	-
SRR22578049	2022	M	Unknown	O unidentifiable:H2	17	CC17	-
SRR6185877	2017	M	Unknown	O unidentifiable:H2	17	CC17	stx2a
SRR4181506	2015	F	Unknown	O103:H2	17	CC17	stx1a
SRR3240984	2014	F	Unknown	O103:H2	17	CC17	stx1a
SRR14208127	2021	M	Unknown	O103:H2	17	CC17	stx1a
SRR7286444	2017	M	Unknown	O103:H2	17	CC17	stx1a
SRR20273441	2022	F	Unknown	O103:H2	17	CC17	stx1a
SRR7282656	2017	F	Morocco	O103:H2	17	CC17	stx1a
SRR22351253	2022	M	Unknown	O103:H2	17	CC17	stx1a
SRR3241842	2014	M	Unknown	O103:H2	17	CC17	stx1a
SRR7286483	2017	F	Italy	O103:H2	17	CC17	stx1a
SRR8740638	2019	M	Unknown	O103:H2	17	CC17	stx1a
SRR9306470	2019	M	Unknown	O103:H2	17	CC17	stx1a
SRR22577994	2022	F	N/A	O103:H2	17	CC17	stx1a
SRR22640875	2022	F	Unknown	O103:H2	17	CC17	stx1a
SRR22640884	2022	F	Unknown	O103:H2	17	CC17	stx1a
SRR22752160	2022	F	Unknown	O103:H2	17	CC17	stx1a
SRR3240977	2014	M	Unknown	O103:H2	17	CC17	stx1a
SRR20508325	2022	M	Unknown	O103:H2	17	CC17	stx1a
SRR20697439	2022	F	Unknown	O103:H2	17	CC17	stx1a
SRR21018068	2022	F	Unknown	O103:H2	17	CC17	stx1a
SRR21525073	2022	F	Spain	O103:H2	17	CC17	stx1a
SRR20429480	2022	M	Italy/Lebanon	O103:H2	17	CC17	stx1a
SRR7277764	2017	F	Unknown	O103:H2	17	CC17	stx1a

SRR7838552	2018	F	N/A	O103:H2	17	CC17	stx1a
SRR7842079	2018	M	Unknown	O103:H2	17	CC17	stx1a
SRR10212219	2019	F	Greece	O103:H2	17	CC17	stx1a
SRR20428648	2022	M	N/A	O103:H2	17	CC17	stx1a
	2018	M	N/A	O103:H2	17	CC17	stx1a
SRR8058698	2018	M	Unknown	O103:H2	17	CC17	stx1a
SRR9873381	2019	M	Mexico	O103:H2	17	CC17	stx1a
SRR9942562	2019	F	Portugal	O103:H2	17	CC17	stx1a
SRR11215181	2020	M	Unknown	O103:H2	17	CC17	stx1a,stx2a
SRR22247567	2022	F	Unknown	O103:H2	17	CC17	stx1a
SRR22398297	2022	M	N/A	O103:H2	17	CC17	stx1a
SRR16941022	2021	F	Unknown	O103:H2	17	CC17	stx1a
SRR18759911	2022	F	Unknown	O103:H2	17	CC17	stx1a
SRR19139386	2022	F	N/A	O103:H2	17	CC17	stx1a
SRR22351259	2022	F	Indonesia	O103:H2	17	CC17	stx1a
SRR3578425	2015	M	Unknown	O103:H2	17	CC17	stx1a
SRR7184203	2017	F	Unknown	O103:H2	17	CC17	stx1a
SRR19347610	2022	F	Unknown	O103:H2	17	CC17	stx1a
SRR19421226	2022	M	Unknown	O103:H2	17	CC17	stx1a
SRR21284101	2022	F	France	O103:H2	17	CC17	stx1a
	2020	F	N/A	O103:H2	17	CC17	stx1a
SRR10212130	2019	F	Unknown	O103:H2	386	CC17	stx1a
SRR3578580	2015	F	N/A	O103:H2	386	CC17	stx1a
